# Mitochondrial Genome Supports Sibling Species of *Angiostrongylus costaricensis* (Nematoda: Angiostrongylidae)

**DOI:** 10.1371/journal.pone.0134581

**Published:** 2015-07-31

**Authors:** Hoi-Sen Yong, Sze-Looi Song, Praphathip Eamsobhana, Share-Yuan Goh, Phaik-Eem Lim, Wan-Loo Chow, Kok-Gan Chan, Elizabeth Abrahams-Sandi

**Affiliations:** 1 Institute of Biological Sciences, University of Malaya, Kuala Lumpur, Malaysia; 2 Chancellery High Impact Research, University of Malaya, Kuala Lumpur, Malaysia; 3 Deaprtment of Parasitology, Faculty of Medicine Siriraj Hospital, Mahidol University, Bangkok, Thailand; 4 Institute of Ocean and Earth Sciences, University of Malaya, Kuala Lumpur, Malaysia; 5 Science Vision Sdn Bhd, Setia Avenue, 33A-4 Jalan Setia Prima S, U13/S, Setia Alam, Seksyen U13, 40170 Shah Alam, Selangor Darul Ehsan, Malaysia; 6 Department of Parasitology, University Costa Rica, San Pedro, Costa Rica; Sichuan University, CHINA

## Abstract

*Angiostrongylus costaricensis* is a zoonotic parasitic nematode that causes abdominal or intestinal angiostrongyliasis in humans. It is endemic to the Americas. Although the mitochondrial genome of the Brazil taxon has been published, there is no available mitochondrial genome data on the Costa Rica taxon. We report here the complete mitochondrial genome of the Costa Rica taxon and its genetic differentiation from the Brazil taxon. The whole mitochondrial genome was obtained from next-generation sequencing of genomic DNA. It had a total length of 13,652 bp, comprising 36 genes (12 protein-coding genes—PCGs, 2 rRNA and 22 tRNA genes) and a control region (A + T rich non-coding region). It is longer than that of the Brazil taxon (13,585 bp). The larger mitogenome size of the Costa Rica taxon is due to the size of the control region as the Brazil taxon has a shorter length (265 bp) than the Costa Rica taxon (318 bp). The size of 6 PCGs and the start codon for ATP6, CYTB and NAD5 genes are different between the Costa Rica and Brazil taxa. Additionally, the two taxa differ in the stop codon of 6 PCGs. Molecular phylogeny based on 12 PCGs was concordant with two rRNA, 22 tRNA and 36 mitochondrial genes. The two taxa have a genetic distance of p = 16.2% based on 12 PCGs, p = 15.3% based on 36 mitochondrial genes, p = 13.1% based on 2 rRNA genes and p = 10.7% based on 22 tRNA genes, indicating status of sibling species. The Costa Rica and Brazil taxa of *A*. *costaricensis* are proposed to be accorded specific status as members of a species complex.

## Introduction

*Angiostrongylus costaricensis* Morera and Céspedes, 1971 is a zoonotic parasitic nematode of the Angiostrongylidae family (superfamily Metastrongyloidea) [1]. It resides in the mesenteric arteries of its definitive hosts and its eggs embryonate in the intestinal wall [1]. There are some 16 definitive hosts, including 10 species of rodents, 1 species of opossum, 2 species of carnivores and 3 species of primates [2]. Dogs have been suggested to play a role as a reservoir host [3].

This parasite was first discovered in humans in Costa Rica [4]. It is endemic to the Americas (from southern USA to northern Argentina in South America) and in humans causes abdominal or intestinal angiostrongyliasis, which mimics appendicitis with eosinophilia [5,6]. The first reported outbreak of abdominal angiostrongyliasis occurred in Guatemala [7]. Databases from 1996 to April 2012 revealed 27 case descriptions of abdominal angiostrongyliasis and 1 case series of 194 patients [8]. The main countries of origin were Costa Rica (89.6%), Brazil (2.7%) and the United States (1.8%). The life cycle of the Brazil taxon of *A*. *costaricensis* in its vertebrate hosts (*Sigmodon hispidus*) and in a mouse model [9,10] is much more complex than that of the Costa Rica taxon [11].

Molecular phylogeny based on COI nucleotide sequences [12] and ITS-2 gene [13] indicated possible occurrence of cryptic species for the *A*. *costaricensis* taxa from Costa Rica and Brazil. A multi-gene phylogeny will help to resolve the taxonomic status.

To date the mitochondrial genome of the Brazil taxon of *A*. *costaricensis* based on long-PCR method has been published [14]. We report here the complete mitochondrial genome (mitogenome) of the Costa Rica taxon of *A*. *costaricensis* by next-generation sequencing and comparison with the mitogenome of Brazil taxon to determine their taxonomic status as sibling species.

## Materials and Methods

### Ethics statement

*A*. *costaricensis* is a parasitic nematode. It is not endangered or protected by law. No permits are required to study this lungworm.

### Specimen and genomic DNA extraction

*A*. *costaricensis* adult specimen preserved in RNAlater (RNA stabilization solution) was a gift from the Department of Parasitology, University of Costa Rica. Whole genomic DNA was extracted using G-spin Total DNA Extraction Mini Kit (iNtRON Biotechnology, Inc, Korea) following the manufacturer’s instructions with minor modification.

### Sample and library preparation

The purified genomic DNA was quantified using Qubit dsDNA HS Assay Kit (Life Technologies, USA) and normalized to 50 ng. Library was prepared using Nextera DNA Sample Preparation Kit (Illumina, USA) following the manufacturer’s protocols. Size estimation of the library was performed on a 2100 Bioanalyzer using High Sensitivity DNA Analysis Kit (Agilent Technologies) and quantified with Qubit 2.0 Fluorometer (Life Technologies, USA).

### Genome Sequencing

The library was normalized to 12 pM and sequenced using the Illumina MiSeq Desktop Sequencer (2 x 150 bp paired-end reads) (Illumina, USA).

### Sequence and genome analysis

Raw sequences were extracted from the Illumina MiSeq system in FASTQ format and the quality of sequences was evaluated using the FastQC software [15]. All the ambiguous nucleotides and reads with an average quality value lower than Q20 were excluded from further analysis. De novo assembly was performed using the CLC Genomic Workbench version 7.0.4 (Qiagen, Germany) and contigs greater than 13 kbp were subjected to BLAST [16] alignment against the nucleotide database at National Center for Biotechnology Information (NCBI). Contigs with hits to mitochondrial genes or genomes were identified and extracted from CLC Genomic Workbench.

### Mitogenome identification, annotation and visualization

A contig identified as mitogenome was manually examined for repeats at the beginning and end of the sequence to establish a circular mtDNA. It was then annotated with MITOS [17] followed by manual validation of the coding regions using the NCBI ORF Finder (http://www.ncbi.nlm.nih.gov/gorf/gorf.html). The sequin file generated from MITOS was edited and submitted to NCBI according to ORF Finder result (NCBI GenBank accession number KR827449). The circular mitogenome of *A*. *costaricensis* was visualized with Blast Ring Image Generator (BRIG) [18].

### Mitogenomes from GenBank

The mitogenome of *A*. *costaricensis* available from GenBank (NC_013067) was based on Brazil taxon. Mitogenomes of *A*. *cantonensis* (NC_013065) and *A*. *vasorum* (NC_018602) were used for comparison with *Aelurostrongylus abstrusus* (NC_019571) as an outgroup.

### Phylogenetic analysis

The total length of the aligned sequences of 36 mt-genes, 12 protein-coding genes (PCGs), 2 rRNA and 22 tRNA genes as well as the selected models used for maximum likelihood (ML) and Bayesian Inference (BI) analyses are summarized in Table 1.

**Table 1:** Information of the aligned sequences of 36 mitochondrial genes, 12 protein-coding genes (PCGs), 2 rRNA and 12 tRNA genes of *Angiostrongylus costaricensis* and related taxa. AIC, Akaike Information Criterion; BIC, Bayesian Information Criterion.

Data set	No. taxa	Total length	Model selected based on AIC	Model selected based on BIC
36 mt-genes	5	13404	GTR+Gamma	J1ef+Gamma
12 PCGs	5	10327	GTR+Gamma	J1ef+Gamma
2 rRNA genes	5	1690	J1+Gamma	J1ef+Gamma
22 tRNA genes	5	1387	J1+Gamma	J1+Gamma

The 12 PCG sequences were separately aligned using ClustalX [19] program and were subsequently edited and trimmed using BioEdit v.7.0.5.3 [20]. The sequences of *rrnS*, *rrnL* and 22 mt-tRNA genes were aligned by MAFFT version 7 [21]. Kakusan v.3 [22] was used to determine the best-fit nucleotide substitution models for maximum likelihood (ML) and Bayesian (BI) analyses selected using the corrected Akaike Information Criterion [23] and the Bayesian Information Criterion [24], respectively. Phylograms of 12 concatenated PCGs, 36 mt-genes, 2 rRNA genes and 22 mt-tRNA genes were constructed using TreeFinder [25]. Bootstrap values (BP) were generated via 1,000 ML bootstrap replicates. Bayesian analyses were conducted using the Markov chain Monte Carlo (MCMC) method via Mr. Bayes v.3.1.2 [26], with two independent runs of 2x10^6^ generations with four chains, and with trees sampled every 200^th^ generation. Likelihood values for all post-analysis trees and parameters were evaluated for convergence and burn-in using the “sump” command in MrBayes and the computer program Tracer v.1.5 (http://tree.bio.ed.ac.uk/software/tracer/). The first 200 trees from each run were discarded as burn-in (where the likelihood values were stabilized prior to the burn-in), and the remaining trees were used for the construction of a 50% majority-rule consensus tree. Phylogenetic trees were viewed and edited by FigTree v.1.4 [27].

## Results

### Mitogenome analysis and features

Next-generation sequencing on Illumina MiSeq Desktop Sequencer generated approximately 4,302,276 reads from *A*. *costaricensis* (Costa Rica taxon) library. Removal of low quality sequence (< Q20) and sequences shorter than 50 nucleotides resulted in 4,190,993 reads. De novo assembly with these reads resulted in 440 contigs with maximum length of 13,652 bp and N50 of 452. The total GC content was 25.1%, with base composition of 26.7% A, 48.2% T, 18.8% G, and 6.3% C.

**Fig 1:**
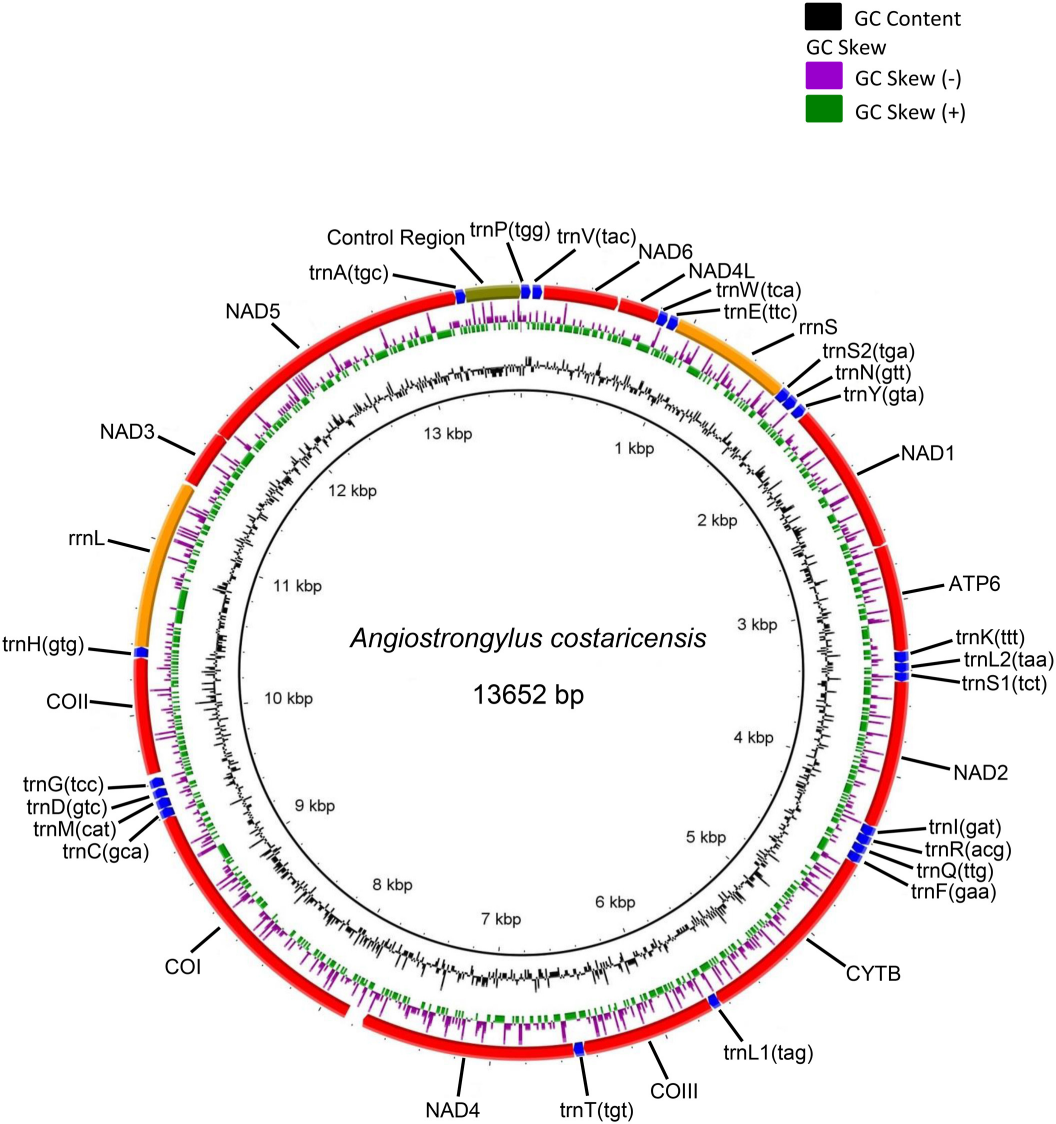
Complete mitogenome of *Angiostrongylus costaricensis* (Costa Rica taxon) with BRIG visualization showing the protein coding genes, rRNAs, tRNAs and non-coding regions. GC skew (0.498) is shown on the outer surface of the ring whereas GC content (25.1%) is shown on the inner surface. The AT skew is -0.287. The intergenic space between NAD4 and COI is 64 bp.

The mitogenome of the Costa Rica taxon of *A*. *costaricensis* was 13,652 bp long, comprising 36 genes (12 protein-coding genes—PCGs, 2 rRNA genes, and 22 tRNA genes) and a non-coding region (A + T-rich control region) (Fig 1, Table 2). All the 36 genes and the control region were located on the H-strand. Spacing sequences were present in 17 regions, ranging from 1 to 64 bp with the largest between NAD4 and COX1 genes. The sequence with 64 bases had clear stem-loop structures and a long poly-T stretch of 11 bp (S1 Fig). Three regions had overlaps with 1 bp each (Table 2). The control region (318 bp) was flanked by trnA(tgc) and trnP(tgg) genes (Fig 1).

**Table 2:** Gene order of mitochondrial genome of *Angiostrongylus costaricensis* (Costa Rica taxon).

Gene	Location	Strand	Size (bp)	Intergenic Sequence	Start/stop codon
trnP(tgg)	1–55	H	55	10	
trnV(tac)	66–121	H	56	9	
NAD6	131–559	H	429	9	ATG/TAG
NAD4L	569–800	H	232		ATT/T
trnW(tca)	801–856	H	56	8	
trnE(ttc)	865–920	H	56	1	
rrnS	922–1618	H	697		
trnS2(tga)	1619–1677	H	59		
trnN(gtt)	1678–1737	H	60	12	
trnY(gta)	1750–1805	H	56		
NAD1	1806–2678	H	873	5	TTG/TAA
ATP6	2684–3292	H	609	1	ATA/TAG
trnK(ttt)	3294–3356	H	63	1	
trnL2(taa)	3358–3412	H	55		
trnS1(tct)	3413–3464	H	52		
NAD2	3465–4311	H	847	1	TTG/T
trnI(gat)	4313–4370	H	58	-1	
trnR(acg)	4370–4426	H	57	2	
trnQ(ttg)	4429–4482	H	54	1	
trnF(gaa)	4484–4539	H	56		
CYTB	4540–5649	H	1110	-1	TTG/TAA
trnL1(tag)	5649–5703	H	55		
COX3	5704–6469	H	766		TTG/T
trnT(tgt)	6470–6527	H	58		
NAD4	6528–7757	H	1230	64	TTG/TAG
COX1	7822–9399	H	1578	-1	ATT/TAA
trnC(gca)	9399–9454	H	56		
trnM(cat)	9455–9514	H	60	3	
trnD(gtc)	9518–9571	H	54	7	
trnG(tcc)	9579–9634	H	56		
COX2	9635–10327	H	693	2	TTG/TAG
trnH(gtg)	10330–10388	H	59		
rrnL	10389–11355	H	967	4	
NAD3	11360–11693	H	334		TTG/T
NAD5	11694–13278	H	1585		ATG/T
trnA(tgc)	13279–13334	H	56		
Control region	13335–13652	H	318		

The commonest start codon was TTG (in 7 PCGs–*cox2*, *cox3*, *cytb*, *nad1*, *nad2*, *nad3*, *nad4*), followed by two each for ATG (*nad5*, *nad6*) and ATT (*cox1*, *nad4l*), and one for ATA (*atp6*). Four PCGs had TAG stop codon (*atp6*, *cox2*, *nad4*, *nad6*) and three had TAA (*cox1*, *cytb*, *nad1*) while the remaining five genes (*cox3*, *nad2*, *nad3*, *nad4l*, *nad5*) had incomplete T stop codon (Table 2).

Table 3 summarizes the nucleotide composition of the mitochondrial whole genome, protein-coding genes, rRNA genes and control region. All were A+T rich. The A + T content for PCGs ranged from 69.5% (*cox1*) to 79.3% (*nad2*). Only one PCG (*cox1*) had A + T content of less than 70%. The A + T content of the non-coding control region was 87.1%. The GC skewness values for the whole genome, PCGs, rRNA genes and control region were positive (0.339 to 0.800) indicating bias toward the use of Gs over Cs. Although the AT skewness value was negative for the whole genome, it was variable for individual genes.

**Table 3:** Nucleotide composition of mitochondrial whole genome, protein-coding genes, rRNA genes and control region of *Angiostrongylus costaricensis*. (a) Costa Rica taxon; (b) Brazil taxon NC_013067.

Region	A%	C%	G%	T%	A+T%	G+C%	AT skew	GC skew
Whole mitogenome	(a) 26.7	6.3	18.8	48.2	74.9	25.1	-0.287	0.498
	(b) 25.4	6.6	20.2	47.8	73.2	26.8	-0.306	0.507
NAD2	(a) 25.8	3.7	17.0	53.5	79.3	20.7	-0.349	0.643
	(b) 22.4	3.4	20.3	53.9	76.3	23.7	-0.413	0.713
COX1	(a) 23.0	8.8	21.7	46.5	69.5	30.5	-0.338	0.423
	(b) 21.7	9.5	23.0	45.8	67.5	32.5	-0.357	0.415
COX2	(a) 25.8	7.4	21.8	45.0	70.9	29.1	-0.271	0.495
	(b) 24.1	6.9	23.8	45.2	69.3	30.7	-0.304	0.550
ATP6	(a) 25.6	5.1	19.5	49.8	75.4	24.6	-0.321	0.585
	(b) 23.3	5.8	22.0	48.8	72.1	27.8	-0.354	0.583
COX3	(a) 24.0	7.2	18.8	50.0	74.0	26.0	-0.351	0.446
	(b) 22.9	7.4	20.6	49.1	71.9	28.1	-0.364	0.470
NAD3	(a) 21.25	2.7	21.25	54.8	76.0	24.0	-0.441	0.773
	(b) 20.5	3.0	22.3	54.2	74.7	25.3	-0.451	0.763
NAD5	(a) 24.5	5.3	18.9	51.3	75.8	24.2	-0.354	0.562
	(b) 25.3	6.3	19.0	49.4	74.7	25.3	-0.323	0.502
NAD4	(a) 26.5	7.7	15.6	50.2	76.7	23.3	-0.309	0.339
	(b) 23.7	8.1	19.2	49.0	72.8	27.2	-0.348	0.408
NAD4L	(a) 23.3	2.2	19.8	54.7	78.0	22.0	-0.403	0.800
	(b) 21.1	2.6	21.6	54.7	75.9	24.1	-0.443	0.788
NAD6	(a) 18.4	4.2	21.2	56.2	74.6	25.4	-0.507	0.669
	(b) 20.4	4.8	20.4	54.4	74.8	25.2	-0.455	0.619
CYTB	(a) 24.4	7.0	19.6	49.0	73.4	26.6	-0.335	0.474
	(b) 22.9	6.8	21.2	49.1	72.0	28.0	-0.364	0.514
NAD1	(a) 23.1	7.5	21.1	48.3	71.5	28.5	-0.352	0.477
	(b) 19.8	7.1	22.9	50.2	70.0	30.0	-0.434	0.527
rrnS	(a) 33.4	7.5	18.2	40.9	74.3	25.7	-0.101	0.416
	(b) 32.9	7.8	18.3	41.0	74.0	26.0	-0.109	0.404
rrnL	(a) 34.3	5.1	15.7	44.9	79.2	20.8	-0.134	0.510
	(b) 32.9	5.7	17.0	44.4	77.3	22.7	-0.149	0.498
Control Region	(a) 42.8	4.1	8.8	44.3	87.1	12.9	-0.017	0.364
	(b) 39.6	6.1	9.8	44.5	84.2	15.8	-0.058	0.234

**Fig 2:**
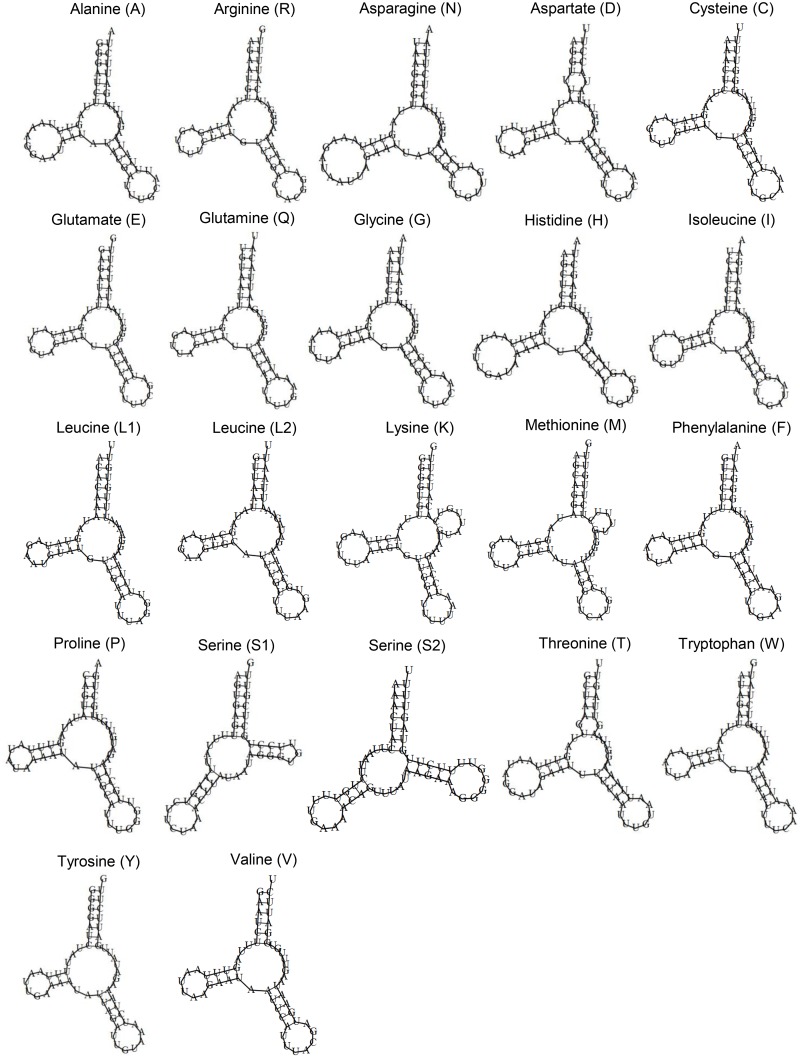
Cloverleaf structure of the 22 inferred tRNAs in the mitogenome of *Angiostrongylus costaricensis* (Costa Rica taxon). The cloverleaf structure lacked the TΨC-arm in 18 tRNAs except serine S1 and serine S2 and the presence of TΨC-loop in lysine and methionine. Serine S1 and serine S2 lacked the DHU-arm.

Of the tRNAs, tryptophan (W) had UCA anticodon (opal suppressor) instead of the canonical Trp tRNA CCA anticodon (Fig 2, Table 2). The cloverleaf structure for 18 tRNAs lacked the TΨC-arm; lysine (K) and methionine (F) lacked the DHU-stem while serine S1 (AGN) and S2 (UCN) had TΨC-arm but lacked the DHU-arm (Fig 2). The number of base pairs in the DHU-stem ranged from 3 to 4 except the absence of DHU-arm in serine S1 and serine S2 (S2 Fig; S1 Table). The TΨC-stem of serine S1 had 4 base pairs and serine S2 had 5 base pairs. The number of bases in the D-loop and TΨC-loop when present was variable (Fig 2).

### Phylogenetic relationships and genetic divergence

**Fig 3:**
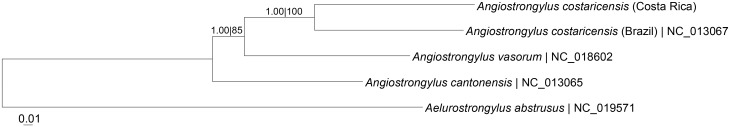
Bayesian inference and maximum likelihood tree based on 12 protein-coding genes of the whole mitogenomes of *Angiostrongylus costaricensis* and congeners with *Aelurostrongylus abstrusus* as outgroup. Numeric values at the nodes are Bayesian posterior probabilities/ML bootstrap.

Fig 3 depicts the molecular phylogeny of *A*. *costaricensis* (Costa Rica taxon) in relation to other taxa of the Angiostrongylidae family based on 12 PCGs. The phylograms based on 36 mt-genes (12 PCGs, 2 rRNA and 22 tRNA genes), two rRNA and 22 tRNA genes were congruent with that based on 12 PCGs (S2–S4 Figs). The *Angiostrongylus* genus was monophyletic, forming a distinct clade from *Ae*. *abstrusus*. The Costa Rica taxon of *A*. *costaricensis* formed a sister group with the Brazil taxon. Table 4 summarizes the genetic divergence of the Costa Rica and Brazil taxa of *A*. *costaricensis* and related taxa of Angiostrongylidae family. The genetic difference (p = 10.7–16.2%) between the Costa Rica and Brazil taxa supports their status as members of a species complex.

**Table 4:** Percentage of uncorrected “p” distance matrix between Costa Rica and Brazil taxa of *Angiostrongylus costaricensis* and related taxa based on (a) 36 mt-genes, (b) 12 protein-coding genes, (c) two rRNA genes, and (d) 22 tRNA genes.

Taxon	1	2	3	4
1. *Angiostrongylus costaricensis* (Costa Rica)	-			
2. *Angiostrongylus costaricensis* (Brazil) NC_013067	(a) 15.3	-		
	(b) 16.2			
	(c) 13.1			
	(d) 10.7			
3. *Angiostrongylus cantonensis* NC_013065	(a) 17.6	(a) 17.4	-	
	(b) 18.1	(b) 18.0		
	(c) 16.3	(c) 15.4		
	(d) 15.4	(d) 15.2		
4. *Angiostrongylus vasorum* NC_018602	(a) 17.9	(a) 17.2	(a) 16.4	-
	(b)18.3	(b) 17.6	(b) 16.8	
	(c) 17.9	(c) 17.3	(c) 16.3	
	(d) 14.4	(d) 13.8	(d) 13.7	
5. *Aelurostrongylus abstrusus* NC_019571	(a) 22.1	(a) 21.8	(a) 20.7	(a) 20.8
	(b) 22.4	(b) 22.1	(b) 21.0	(b) 21.0
	(c) 21.9	(c) 21.2	(c) 19.6	(c) 21.0
	(d) 19.4	(d) 20.3	(d) 18.0	(d) 18.8

## Discussion

Mitochondrial genomes of nematodes have been studied with respect to genetics, epidemiology, systematics, phylogeny and evolution [28–34]. To date, of the 13 species of *Angiostrongylus* genus [35] the mitogenomes of three species (*A*. *cantonensis*, *A*. *costaricensis* Brazil taxon, and *A*. *vasorum*) have been documented [14,31,36]. Additionally the mitogenome of *Aelurostrongylus abstrusus*, another member of the Angiostrongylidae has been published [37]. All these mitogenomes were sequenced by the long-PCR method. The present mitogenome of *A*. *costaricensis* (Costa Rica taxon) was obtained by next-generation sequencing.

Based on COI nucleotide sequences the Costa Rica taxon of *A*. *costaricensis* was quite different from the Brazil taxon, with an uncorrected p-distance of 11.4% [12]. Similar differentiation has been reported for the nuclear ITS-2 gene [13]. The COI and ITS-2 results indicated the possible occurrence of sibling species for the *A*. *costaricensis* taxa.

The mitogenome size of *A*. *costaricensis* (Costa Rica taxon) (13,652 bp) is larger than those of the Brazil taxon (13,585 bp) and the congeners *A*. *cantonensis* (13,491–13,502 bp) as well as *A*. *vasorum* (13,422 bp) but is smaller than that of *Ae*. *abstrusus* (13,913 bp). The larger mitogenome size of the Costa Rica taxon (*A*. *costaricensis*) than the Brazil taxon is due to the size of the control region as the Brazil taxon has a shorter length (265 bp) than the Costa Rica taxon (318 bp). The long intergenic sequence between *nad4* and *cox1* genes has 64 bp with a 11-bp poly-T stretch in the Costa Rica taxon compared to 75 bp with poly-T stretch not exceeding 5 bp in the Brazil taxon. Overlaps are not present in the Brazil taxon but 3 occur in the Costa Rica taxon (S2 Table).

With the exception of COX1-3, NAD1, NAD4L and NAD5 genes, the sizes of the other 6 PCGs are different between the Costa Rica and Brazil taxa of *A*. *costaricensis* (Table 5). The start codon for ATP6, CYTB and NAD5 genes in the Costa Rica taxon differs from those of the Brazil taxon. Additionally, the two taxa differ in the stop codon of 6 PCGs (*cox2*, *cytb*, *nad1*, *nad2*, *nad3* and *nad4*) (Table 5). The Costa Rica taxon has 5 incomplete T stop codons (*cox3*, *nad2*, *nad3*, *nad4l*, *nad5*) compared to 3 in the Brazil taxon (*cox3*, *nad4l*, *nad5*). The incomplete T stop codons can be converted to TAA by post-translational polyadenylation [38].

**Table 5:** Start and stop codons and size of mitochondrial protein-coding genes of Brazil (NC_013067) and Costa Rica taxa of *Angiostrongylus costaricensis*.

Gene	Costa Rica Start/Stop codon	Costa Rica Size (bp)	Brazil Start/Stop codon	Brazil Size (bp)
*atp6*	ATA/TAG	609	ATT/TAG	600
*cox1*	ATT/TAA	1578	ATT/TAA	1578
*cox2*	TTG/TAG	693	TTG/TAA	693
*cox3*	TTG/T	766	TTG/T	766
*cytb*	TTG/TAA	1111	ATG/TAG	1101
*nad1*	TTG/TAA	873	TTG/TAG	873
*nad2*	TTG/T	832	TTG/TAA	849
*nad3*	TTG/T	334	TTG/TAG	336
*nad4*	TTG/TAG	1227	TTG/TAA	1230
*nad4l*	ATT/T	232	ATT/T	232
*nad5*	ATG/T	1585	ATA/T	1582
*nad6*	ATG/TAG	438	ATG/TAG	432

Of the 22 tRNAs, 17 have different length between the Costa Rica and Brazil taxa (Table 6). The total length of the tRNAs for the Costa Rica taxon (1247 bp) is longer than that for the Brazil taxon (1230 bp). The cloverleaf structure for the tRNAs is similar for both taxa.

**Table 6:** Anticodon and length of tRNAs of Costa Rica and Brazil (NC_013067) taxa of *Angiostrongylus costaricensis*. W(Trp) had UCA anticodon (opal nonsense suppressor) instead of canonical CCA anticodon.

tRNA	Costa Rica anticodon	Costa Rica length	Brazil anticodon	Brazil length
P(Pro)	UGG	55	UGG	54
V(Val)	UAC	56	UAC	54
W(Trp)	UCA	56	UCA	59
E(Glu)	UUC	56	UUC	59
S2(Ser)	UGA	59	UGA	52
N(Asn)	GUU	60	GUU	60
Y(Tyr)	GUA	56	GUA	55
K(Lys)	UUU	63	UUU	60
L2(Leu)	UAA	55	UAA	56
S1(Ser)	UCU	52	UCU	52
I(Ile)	GAU	58	GAU	54
R(Arg)	ACG	57	ACG	51
Q(Gln)	UUG	54	UUG	55
F(Phe)	GAA	56	GAA	57
L1(Leu)	UAG	55	UAG	56
T(Thr)	UGU	58	UGU	58
C(Cys)	GCA	56	GCA	56
M(Met)	CAU	60	CAU	59
D(Asp)	GUC	54	GUC	54
G(Gly)	UCC	56	UCC	58
H(His)	GUG	59	GUG	56
A(Ala)	UGC	56	UGC	55

The A+T content of the whole mitogenome in the Costa Rica taxon of *A*. *costaricensis* (74.9%) is higher than that of the Brazil taxon (73.2%) (Table 3). The control region, rRNA genes and all the PCGs (excepting *nad6*) in the Costa Rica taxon have higher A + T content (Table 3).

In the present study based on 36 mt-genes, 12 PCGs, 2 rRNA and 22 tRNA genes the Costa Rica taxon of *A*. *costaricensis* is distinctly different from the Brazil taxon, with uncorrected genetic distance of p = 15.3%, 16.2%, 13.1% and 10.7%, respectively (Table 4). The genetic distances are comparable to those of other nematode cryptic species. Based on COI sequences, the genetic distance bewteen the sibling species *A*. *cantonensis* and *A*. *malaysiensis* is p = 11.1–11.7% [8]. The interspecific divergence bewteen *A*. *cantonensis* and *A*. *malaysiensis* is p = 1.7–4.1% based on 66 kDa protein gene [39] and p = 8.3–9.2% based on CYTB sequences [36]. In contrast, based on CYTB gene the intraspecific divergence for *A*. *cantonensis* is p = 0–2.9% and for *A*. *malaysiensis* p = 0–0.1% [40]. In the *Rhabditis* (*Pellioditis*) *marina* species complex, the three cryptic species have average interspecific divergence (based on COI gene) of 4.6–11.7% [41]. The present findings of genetic distance of p = 10.7–16.2% (Table 4) between the Costa Rica and Brazil taxa of *A*. *costaricensis* provide strong molecular evidence for them to be treated as members of a species complex, as earlier indicated by COI sequences [12]. In that case, in accordance with the type locality the Costa Rica taxon belongs to the nominal species. The Brazil taxon therefore warrants a new specific name.

In summary, we have successfully sequenced the whole mitochondrial genome of the Costa Rica taxon of *A*. *costaricensis* by next-generation sequencing. The genome features are similar to other Angiostrongylidae taxa. The phylogenetic tree based on 36 mt-genes is concordant with those based on 12 PCGs, 2 rRNA and 22 tRNA genes. Based on distinct genetic difference, the Costa Rica and Brazil taxa of *A*. *costaricensis* are proposed to be accorded specific status as members of a species complex.

## Supporting Information

S1 Fig Secondary structure of 64 bp intergenic sequence of *Angiostrongylus costaricensis* (Costa Rica taxon) mitogenome. (TIF)

S2 Fig Bayesian inference and maximum likelihood tree based on 36 mitochondrial genes of the whole mitogenomes of *Angiostrongylus costaricensis* and congeners with *Aelurostrongylus abstrusus* as outgroup. Numeric values at the nodes are Bayesian posterior probabilities/ML bootstrap.
(TIF)

S3 Fig Bayesian inference and maximum likelihood tree based on *rrnL* and *rrnS* genes of the whole mitogenomes of *Angiostrongylus costaricensis* and congeners with *Aelurostrongylus abstrusus* as outgroup. Numeric values at the nodes are Bayesian posterior probabilities/ML bootstrap.
(TIF)

S4 Fig Bayesian inference and maximum likelihood tree based on 22 tRNA genes of the whole mitogenomes of *Angiostrongylus costaricensis* and congeners with *Aelurostrongylus abstrusus* as outgroup. Numeric values at the nodes are Bayesian posterior probabilities/ML bootstrap.
(TIF)

S1 Table Number of base pairs in DHU-stem and TΨC-stem of mt-tRNAs of *Angiostrongylus costaricensis* (Costa Rica taxon). (DOCX)

S2 Table Size (bp) of intergenic sequence in the Brazil (NC_013067) and Costa Rica taxa of *Angiostrongylus costaricensis*. (DOCX)
