# Supplementary figures and images for: Mitochondrial Genome Supports Sibling Species of Angiostrongylus costaricensis (Nematoda: Angiostrongylidae)
Source: PLoS One. 2015 Jul 31;10(7):e0134581. doi: 10.1371/journal.pone.0134581 (PMC4521872; doi:10.1371/journal.pone.0134581)

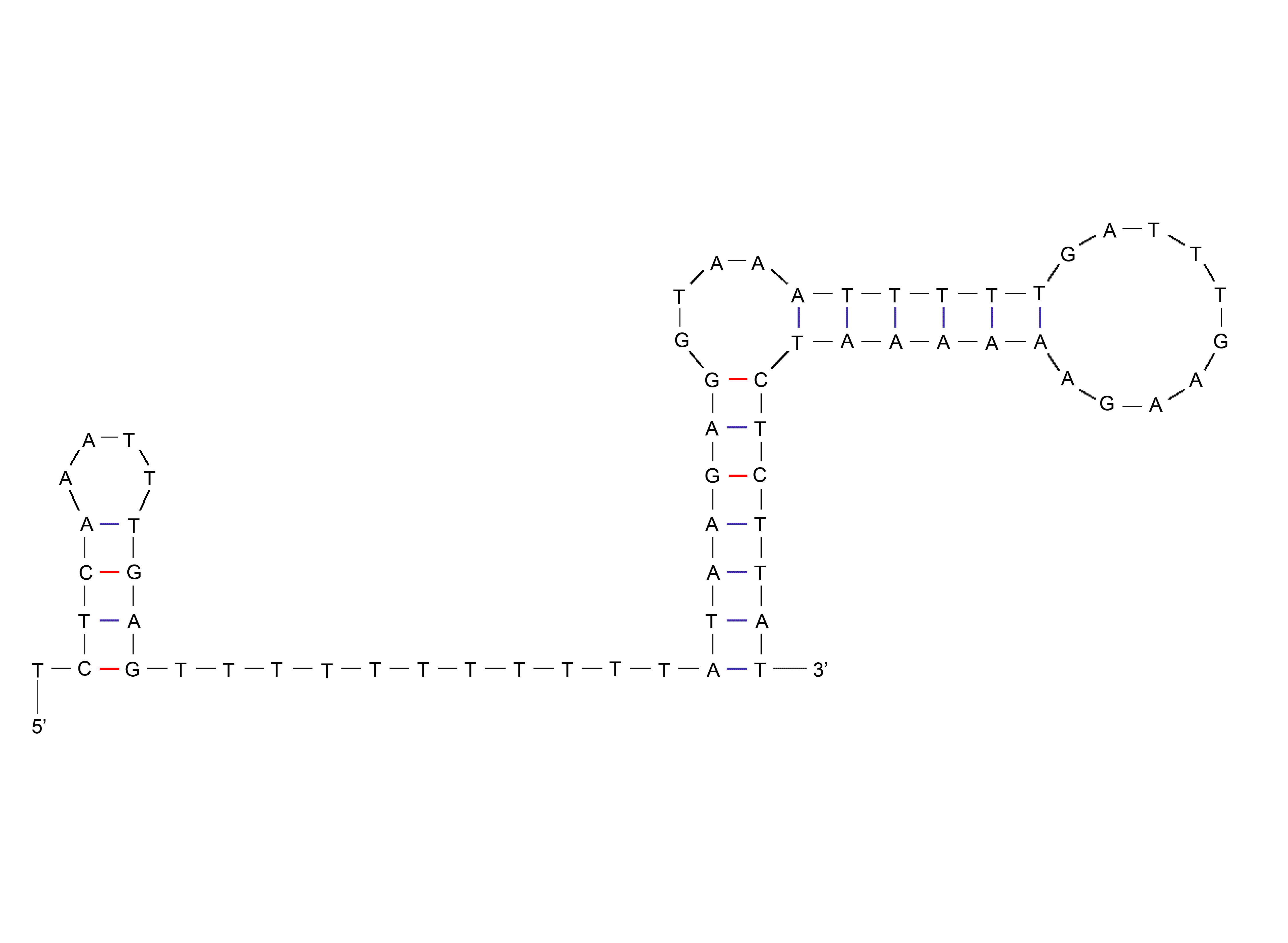

Supplement: S1 Fig — (TIF) [file pone.0134581.s001.tif]

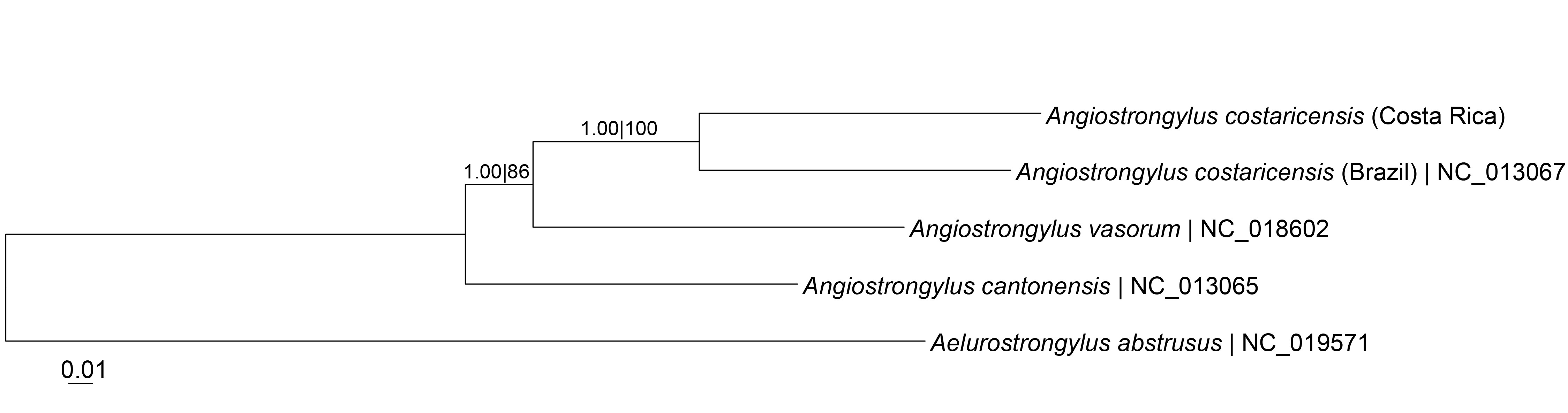

Supplement: S2 Fig — Numeric values at the nodes are Bayesian posterior probabilities/ML bootstrap. (TIF) [file pone.0134581.s002.tif]

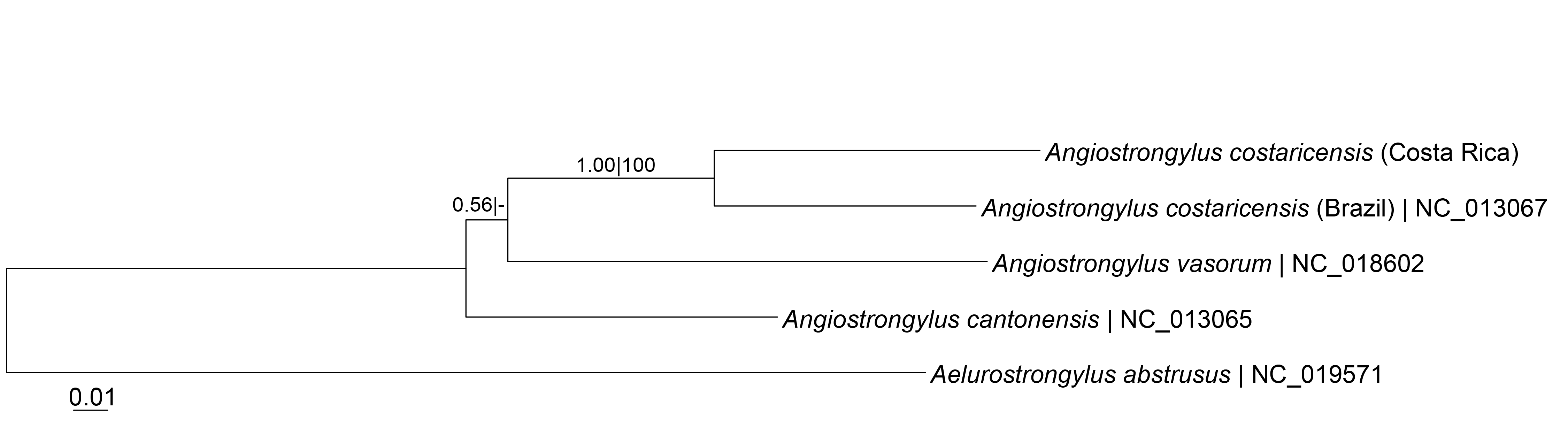

Supplement: S3 Fig — Numeric values at the nodes are Bayesian posterior probabilities/ML bootstrap. (TIF) [file pone.0134581.s003.tif]

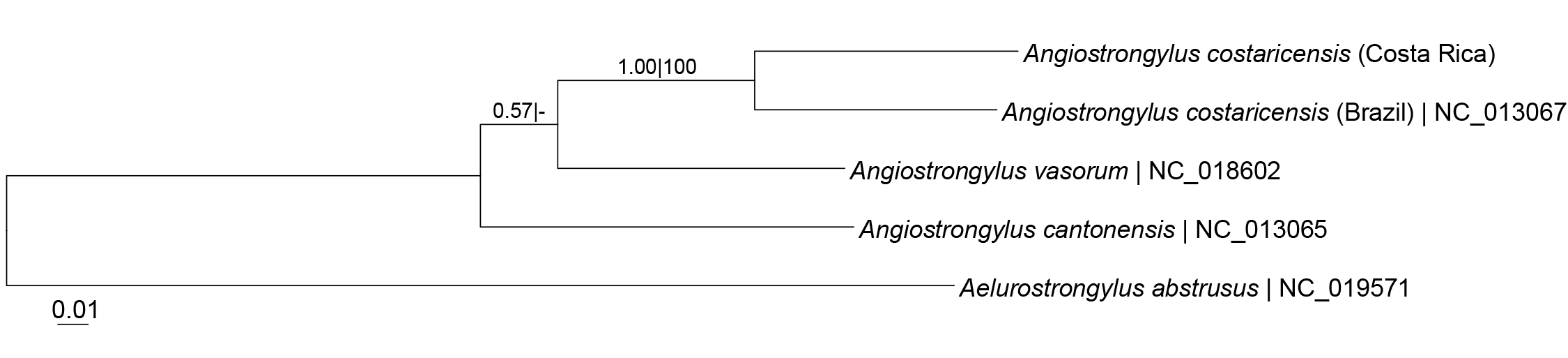

Supplement: S4 Fig — Numeric values at the nodes are Bayesian posterior probabilities/ML bootstrap. (TIF) [file pone.0134581.s004.tif]
