# Supplementary material for: Mitochondrial Genome Supports Sibling Species of Angiostrongylus costaricensis (Nematoda: Angiostrongylidae)
Source: PLoS One. 2015 Jul 31;10(7):e0134581. doi: 10.1371/journal.pone.0134581 (PMC4521872; doi:10.1371/journal.pone.0134581)
Supplement: S1 Table — (DOCX) [file pone.0134581.s005.docx]

**S1 Table. Number of base pairs in DHU-stem and TΨC-stem of mt-tRNAs of *Angiostrongylus costricensis* (Costa Rica taxon).** absent, lack entire arm; #, only TΨC-loop present.

| tRNA | DHU-stem | TΨC-stem |
| --- | --- | --- |
| Alanine | 3 | absent |
| Arginine | 4 | absent |
| Asparagine | 4 | absent |
| Aspartate | 4 | absent |
| Cysteine | 4 | absent |
| Glutamate | 4 | absent |
| Glutamine | 4 | absent |
| Glycine | 4 | absent |
| Histidine | 4 | absent |
| Isoleucine | 4 | absent |
| Leucine L1 (CUN) | 4 | absent |
| Leucine L2 (UUR) | 4 | absent |
| Lysine | 4 | # |
| Methionine | 4 | # |
| Phenyalanine | 4 | absent |
| Proline | 4 | absent |
| Serine S1 (AGN) | absent | 4 |
| Serine S2 (UCN) | absent | 5 |
| Threonine | 4 | absent |
| Tryptophan | 4 | absent |
| Tyrosine | 3 | absent |
| Valine | 4 | absent |
