# Supplementary material for: Mitochondrial Genome Supports Sibling Species of Angiostrongylus costaricensis (Nematoda: Angiostrongylidae)
Source: PLoS One. 2015 Jul 31;10(7):e0134581. doi: 10.1371/journal.pone.0134581 (PMC4521872; doi:10.1371/journal.pone.0134581)
Supplement: S2 Table — (DOCX) [file pone.0134581.s006.docx]

**S2 Table. Size (bp) of intergenic sequence in the Brazil (NC_013067) and Costa Rica taxa of *Angiostrongylus costaricensis.***

| Gene | Costa Rica | Brazil |
| --- | --- | --- |
| trnP(Pro) | 10 | 2 |
| trnV(Val) | 9 | 8 |
| NAD6 | 9 |  |
| NAD4L |  |  |
| trnW(Trp) | 8 | 7 |
| trnE(Glu) | 1 |  |
| rrnS |  | 1 |
| trnS2(Ser) |  |  |
| trnN(Asn) | 12 | 4 |
| trnY(Tyr) |  |  |
| NAD1 | 5 | 15 |
| ATP6 | 1 | 1 |
| trnK(Lys) | 1 | 1 |
| trnL2(Leu) |  |  |
| trnS1(Ser) |  |  |
| NAD2 | 1 | 7 |
| trnI(Ile) | -1 |  |
| trnR(Arg) | 2 | 2 |
| trnQ(Gln) | 1 | 2 |
| trnF(Phe) |  | 8 |
| CYTB | -1 |  |
| trnL1(Leu) |  |  |
| COX3 |  |  |
| trnT(Thr) |  |  |
| NAD4 | 64 | 75 |
| COX1 | -1 |  |
| trnC(Cys) |  |  |
| trnM(Met) | 3 | 1 |
| trnD(Asp) | 7 | 3 |
| trnG(Gly) |  |  |
| COX2 | 2 | 6 |
| trnH(His) |  |  |
| rrnL | 4 |  |
| NAD3 |  | 13 |
| NAD5 |  |  |
| trnA(Ala) |  |  |
| Control region |  |  |
